# Accurate Atomic-Scale Imaging of Two-Dimensional Lattices Using Atomic Force Microscopy in Ambient Conditions

**DOI:** 10.3390/nano12091542

**Published:** 2022-05-02

**Authors:** Sunghyun Kim, Donghyeon Moon, Bo Ram Jeon, Jegyeong Yeon, Xiaoqin Li, Suenne Kim

**Affiliations:** 1 Department of Applied Physics, Hanyang University, Ansan 15588, Korea; shdak6990@gmail.com (S.K.); brhu1018@gmail.com (B.R.J.); jaekyeong.yeon@gmail.com (J.Y.); 2 Department of Photonics and Nanoelectronics, Hanyang University, Ansan 15588, Korea; dhm227@hanyang.ac.kr; 3 Center for Complex Quantum Systems, Department of Physics, The University of Texas at Austin, Austin, TX 78712, USA; elaineli@physics.utexas.edu; 4 Texas Materials Institute, The University of Texas at Austin, Austin, TX 78712, USA

**Keywords:** AFM, TMD, LFM, graphene, transition metal dichalcogenides, atomic-scale imaging

## Abstract

To facilitate the rapid development of van der Waals materials and heterostructures, scanning probe methods capable of nondestructively visualizing atomic lattices and moiré superlattices are highly desirable. Lateral force microscopy (LFM), which measures nanoscale friction based on the commonly available atomic force microscopy (AFM), can be used for imaging a wide range of two-dimensional (2D) materials, but imaging atomic lattices using this technique is difficult. Here, we examined a number of the common challenges encountered in LFM experiments and presented a universal protocol for obtaining reliable atomic-scale images of 2D materials under ambient environment. By studying a series of LFM images of graphene and transition metal dichalcogenides (TMDs), we have found that the accuracy and the contrast of atomic-scale images critically depended on several scanning parameters including the scan size and the scan rate. We applied this protocol to investigate the atomic structure of the ripped and self-folded edges of graphene and have found that these edges were mostly in the armchair direction. This finding is consistent with the results of several simulations results. Our study will guide the extensive effort on assembly and characterization of new 2D materials and heterostructures.

## 1. Introduction

In recent years, atomically thin layers and heterostructures of van der Waals (vdW) materials prepared via chemical vapor deposition (CVD) or mechanical exfoliation/stacking have been intensively studied because of their unique properties and potential applications in quantum electronics and nanophotonics [1,2,3,4]. By changing the number of layers or controlling the twist angle between adjacent layers [5,6,7], remarkable quantum phases and properties have been discovered, including unconventional superconductivity [8,9], Hofstadter’s butterfly effect [10,11,12], Mott transition in graphene bilayers [7,13], and quantized exciton states in moiré crystals formed by twisting transition metal dichalcogenides (TMD) bilayers [14,15,16,17]. However, wrinkles and bubbles inevitably exist in stacked vdW bilayers [18,19,20], causing undesirable spatial variations in strains and disorder in moiré superlattices. Ultrafast laser nondestructive technology allows the study of strain, stress, and structural properties currently on the scale of tens to hundreds of nanometers [21,22,23,24]. The possibility of imaging atomic-scale strain-induced lattice distortion [25] or even controlling the twist angle using scanning probe methods [10] has been demonstrated. Nevertheless, such experiments remain very challenging in general.

Different atomic-scale microscopy techniques provide complementary information and capabilities. Scanning tunneling microscopy (STM) is capable of obtaining information on electronic band structures but requires a conducting substrate and ultra-high vacuum environment. Transmission electron microscopy (TEM) offers chemical identification capabilities. However, TEM experiments require an elaborate sample preparation process [26,27] and often damage the two-dimensional (2D) layers during imaging [28]. Lateral force microscopy (LFM) is an atomic force microscopy (AFM)-based technique operating in the contact mode. It does not require a conducting substrate or time-consuming sample preparation procedures. Such flexibility makes LFM a versatile tool capable of characterizing nondestructively a wide range of 2D materials and nanostructures under ambient conditions [29,30,31,32,33,34].

A number of challenges, however, have prevented the wide application of LFM in obtaining accurate images of the atomic lattices of 2D materials routinely [35,36,37]. LFM images are often distorted by thermal drift and less-than-optimal scan parameters. In addition, the authenticity of filtered LFM images (such as inverse fast Fourier transformation (FFT) images) is questionable, if the quality of the original image is poor or the filtering procedures are not executed with great care [38]. Contact-mode atomic lattice images of 2D materials acquired in air have only been reported by a few groups worldwide [32,33,39,40,41,42,43,44,45,46,47,48,49,50,51], and most of them were obtained using special homebuilt or symmetrically-designed AFMs [28,45,46,47,48,49], functionalized tips [50], or carbon nanotube tips [51] (Table S1). These specialized approaches are difficult to adopt.

In this paper, we demonstrated a protocol capable of obtaining atomic lattice images of 2D materials by LFM under ambient conditions. The protocol is based on an in-depth understanding of how feedback loops of an AFM and various parameters such as scan rate, scan size, and gain can influence the LFM raw (unflatten and unfiltered) images in the presence of drifts. In addition, we discussed the effects of load and substrate roughness on the accuracy and sharpness of atomic lattice images of 2D materials. We drew examples from TMD monolayers (MoS_2_ and MoSe_2_) and graphene. By selecting appropriate scan parameters, the geometric distortions in the LFM raw images decreased, and the signal-to-noise ratio in the raw FFT images improved significantly. The protocol allows one to successfully identify and interpret the crystallographic structures of the torn and self-folded edges of graphene. This demonstrated that this protocol can be used successfully to determine the crystalline axes of folded graphene nanoribbons.

## 2. Materials and Methods

### 2.1. Sample Preparation

We purchased a piece of SiO_2_/Si wafer covered with triangular MoS_2_ monolayers (6carbon Technology: Shenzhen, China). Graphene and MoSe_2_ were prepared by exfoliation using a scotch tape. Each sample thus prepared was attached to a metal disk with a double-sided carbon tape. The carbon tape was squeezed to minimize the viscoelastic behavior of the polymer adhesive to avoid associated imaging artifacts [52,53].

### 2.2. LFM

The LFM technique is commonly used to measure the friction between the AFM tip and the sample by recording the lateral deflection signal from the backside of the cantilever. The higher the friction, the more the torsion of the cantilever. An image with 128 × 128 pixels was obtained from a clean Si sample without moving the AFM tip (i.e., the zero scan) to evaluate the noise floor. The RMS roughness of this image, calculated using XEI version 4.3.0 (Park Systems, Suwon, Korea), was ~38 pm for the main NX 10 AFM (Park Systems, Suwon, Korea). A noise floor between 30 and 60 pm is considered typical [54]. 

We obtained the atomic-scale LFM images of TMDs (MoSe_2_ or MoS_2_) and multilayer graphene with a Si cantilever (NSC36) under ambient conditions (13.1–19.6 °C; relative humidity: 12–20%) using the NX10 AFM. The NSC36 series probes (MikroMasch, Tallinn, Estonia) have three beam-shaped cantilevers with different spring constants on one side of the holder chip. Usually, we use the cantilever with the lowest normal spring constant (NSC36C) among them: the lower the normal spring constant of the rectangular cantilever is, the better the lateral force difference can be detected. More precisely, the force sensitivity of the LFM is determined by the torsional spring constant, which is proportional to the normal spring constant for a given rectangular cantilever geometry [55,56,57,58]. The LFM images of graphene were acquired at 26 ± 1 °C and an RH of 59% ± 2% using another cantilever (also NSC36C) and a different AFM system (XE-100; Park Systems). The XE-100 AFM was operated in the low-voltage mode for the highest-resolution imaging. When operating the NX10 AFM, we used a standard 50 μm XY-scanner, which required additional calibration steps. Highly Ordered Pyrolytic Graphite (HOPG) was used to calibrate the scanner. All atomic lattice images presented here were acquired without closed-loop control. Prior to collecting the atomic-scale LFM data from each sample, the sample surface was locally cleaned several times using the same AFM tip (typically, an area of 20 μm^2^ at a scan rate of 7 Hz and a load of about 10 nN) [59,60,61,62,63]. 

We used the Cleveland method to calibrate the spring constant [64]. The normal spring constants of the NSC36C cantilevers used for our atomic-scale imaging ranged from 0.77 to 1.43 Nm^−1^. For the cantilever with a normal spring constant of 1.43 Nm^−1^ (having the lowest LFM force sensitivity among the cantilevers used in our experiments), the torsional spring constant calculated with the dimensions provided by the company was 14.87–23.01 Nm^−1^ (the range results from the uncertainty of the given tip height) and the lateral optical lever sensitivity obtained from the NX10 following the wedge method using the NT-MDT TGG1 silicon grating was 80.9–112.8 nNV^−1^ [58,65]. In addition, we expected the tip stiffness to be larger than 100 Nm^−1^, since the smallest radius of the curvature measured in the experiment is approximately 52.3 nm [66]. The contact stiffness between the 1.43 Nm^−1^ Si tip and the reference Si substrate at the load of 10.3 nN (RH: 23% ± 2%) was estimated to be 240.9 Nm^−1^ according to the Hertz model when the tip was medium sharp (See Supplementary Materials for detailed calculations; Figure S1) [67,68,69]. The applied normal force was between 2.9 and 33.4 nN.

The color bars of all LFM images represent the voltage reading from the photodiode (C–D), which was proportional to the friction at the tip−sample contact [70]. The lattice−scale contrast came from the well-known stick-slip phenomenon [50]. Brighter locations in the LFM images corresponded to the centers of the hexagonal rings (hollow sites) in the honeycomb lattice [71]. 

Black bands appear on the left of the atomic-scale images, when the color range was adjusted. The origin of the bands is initial sticking [50]. Figure S2 shows a MoS_2_ trace image before and after the color range adjustment. To visualize the atomic lattice structure clearly without these black bands, we recommend applying a minimum of 20% overscan (the overscan is an overscanned area that is not used for sampling image data) when acquiring images with a scan size of less than 5 nm. We applied 5−20% overscan throughout the work. 

### 2.3. FFT

We used Gwyddion software from http://gwyddion.net/ (accessed on 10 April 2022) for our analysis. We performed 2D FFT to analyze the atomic-scale LFM images of MoSe_2_, graphene, and MoS_2_. The inverse FFT images accurately revealed the respective honeycomb lattices and yielded the expected lattice constants of MoSe_2_, graphene, and MoS_2_.

## 3. Results and Discussion

### 3.1. Accurate Atomic Lattice Imaging Protocol

#### 3.1.1. Effect of the XY Feedback, Scan Size, Scan Rate, Gain, Load, and Substrate Roughness

##### Effect of the XY Feedback

**Figure 1:**
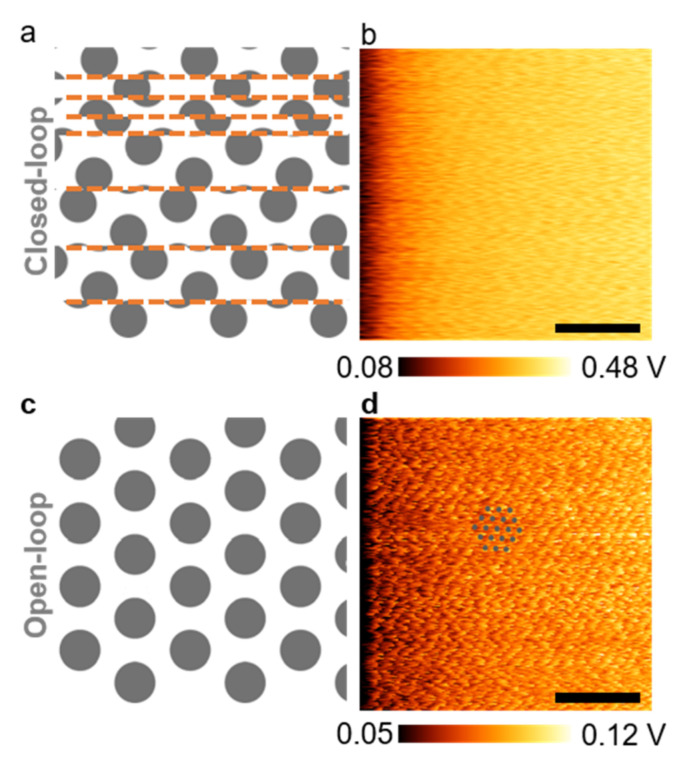
Lateral force microscopy (LFM) raw images obtained with or without engaging the XY closed-loop control in air (color range adjusted). (**a**) Schematic representation of a jumbled LFM image due to the use of a XY closed-loop control. The orange dashed lines indicate the locations at which the piezo stage was abruptly repositioned due to the XY closed-loop control; (**b**) LFM image of a MoS_2_ monolayer obtained with a closed-loop control (calibrated scale bar: 2 nm; scan rate: 21 Hz); (**c**) schematic illustration of the LFM image of a two-dimensional (2D) lattice expected in the absence of the XY closed-loop control; (**d**) LFM image of a MoS_2_ monolayer obtained without a closed-loop control, which revealed a periodic pattern from the atomic lattice (calibrated scale bar: 2 nm; scan rate: 21 Hz). The grey dots are a guide for the eye and correspond to the lattice of rate MoS_2_.

We discussed several critical AFM parameters that influenced the quality of LFM images of 2D atomic lattices sequentially. First, the XY position feedback loop needed to be disabled to visualize the atomic lattices in air. This rather counterintuitive finding is due to the slow and small spatial drift of the tip relative to the sample caused by ambient temperature fluctuations. The XY closed-loop system typically compensated for nanometer-level drifts [72,73], but not the drifts in the picometer-to-subangstrom range required for atomic-scale imaging. In addition, open-loop scans (without XY position feedback) showed less noise than closed-loop scans [74]. When the XY feedback was turned on, the associated sensor noise and a sudden nanometer-level compensational reposition of the piezo actuator inevitably introduced irregularity to an image, as illustrated in Figure 1a. An example of a closed-loop LFM image of MoS_2_ is presented in Figure 1b, which failed to reveal any information related to the lattice structure. In contrast, such irregularity was eliminated in an open-loop scan as illustrated in Figure 1c and as demonstrated in the LFM image of the same MoS_2_ monolayer (Figure 1d). All parameters used for obtaining Figure 1b,d were the same, except for the setting of the XY closed-loop feedback. A periodic pattern related to the underlying lattice is only visible in Figure 1d.

##### Scan Size Dependence

**Figure 2:**
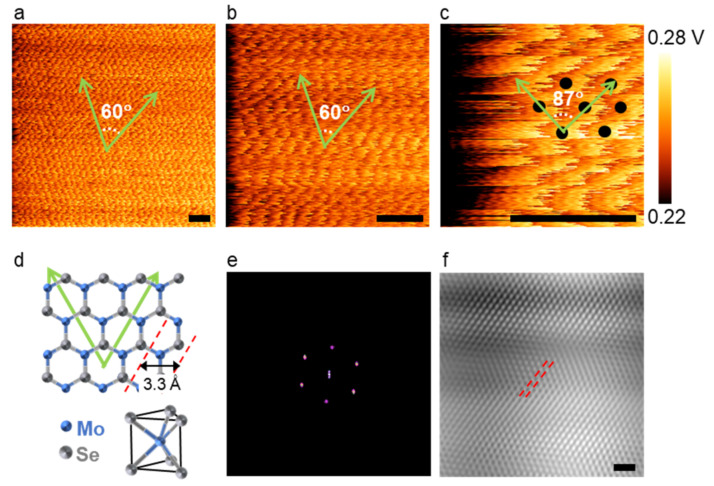
Scan size-dependent atomic lattice images (only color range adjusted). (**a**–**c**) LFM raw images of a MoSe_2_ monolayer acquired with nominal lateral scan sizes of 160, 70, and 25 Å, respectively (calibrated scale bar: 1 nm; scan rate: 19 Hz). All green arrows are aligned along the zigzag direction as illustrated in (**d**). The black dots in (**c**) are for visual guidance and represent the centers of the hexagonal rings in MoSe_2_ (i.e., the hollow sites); (**d**) schematic model of MoSe_2_. Mo and Se atoms are marked with blue and grey spheres, respectively; (**e**) filtered FFT image of the LFM raw image shown in (**a**); (**f**) inverse FFT image (scale bar: 1 nm).

Secondly, whether the LFM images can correctly represent the atomic lattices depends on the scan size. One might expect that taking an image with a smaller scan size is advantageous in revealing atomic-scale details. We have found that images were distorted if the scan size was too small. We presented LFM images taken from a MoSe_2_ layer with lateral scan sizes of 160, 70, and 25 Å at 19 Hz (Figure 2a–c). In all these three images, periodic patterns are evident. We have drawn two green arrows along the pixels of the same brightness in the LFM images. These arrows are aligned with the two nearest zigzag directions of the MoSe_2_ hexagonal lattice [75,76], as illustrated in Figure 2d. The two arrows were expected to make an angle of 60°. While this angle was measured as 60° ± 1° for scan sizes of 160 Å (Figure 2a) and 70 Å (Figure 2b), the analysis of Figure 2c yielded an angle of 87° ± 1°, which clearly deviated from the expected value. In fact, the type of skewed lattice shown in Figure 2c was frequently observed in many scanning probe microscopy images beyond LFM images. We further analyzed the LFM data shown in Figure 2a to extract the lattice constant of MoSe_2_. We presented a filtered FFT image of Figure 2a and the inverse FFT (the filtered real-space image) in Figure 2e,f, respectively. The spacing between the red diagonal lines in Figure 2f was measured to be ~3.3 Å, which agreed well with the previously reported lattice constant of MoSe_2_ in the range of 3.288–3.320 Å [77,78]. Thus, we concluded that the FFT bright spots in Figure 2e are due to the periodic lattice structure of MoSe_2_ and the original LFM image presented in Figure 2a represents the MoSe_2_ lattice structure with high accuracy.

##### Scan Rate Dependence

**Figure 3:**
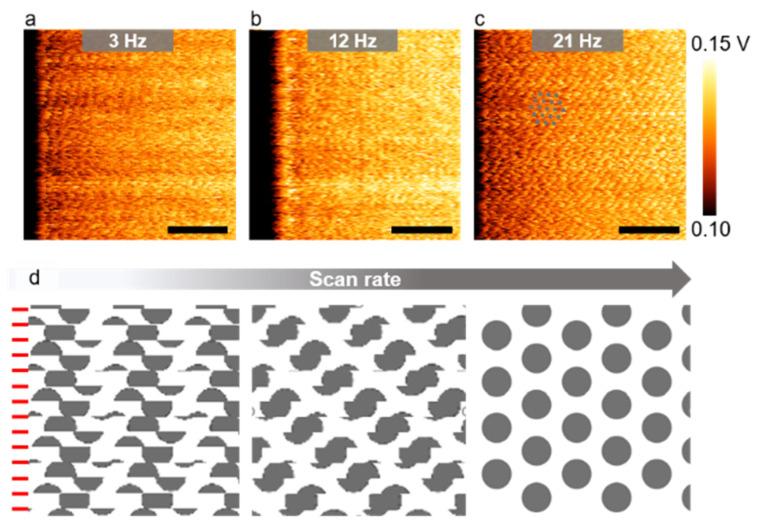
Scan rate-dependent atomic lattice images (color range adjusted). (**a**–**c**) LFM raw images of MoS_2_ acquired at various scan rates. Grey dots are a guide for the eye and correspond to the centers of the MoS_2_ hexagons. All scale bars are calibrated and correspond to 2 nm; (**d**) schematic representations of the LFM images as a function of the scan rate, assuming a constant drift velocity. The red lines mark horizontal scan lines. Image distortion is reduced with an increasing scan rate.

Thirdly, the scan rate should be set to approximately an order of magnitude larger than the commonly used values to image the atomic lattices accurately. Several atomic-level LFM images of a MoS_2_ monolayer acquired at 3, 12, and 21 Hz without closed-loop control are displayed in Figure 3a–c, respectively. Except for the scan rate, all parameters used for obtaining Figure 3a–c were the same. The features associated with the MoS_2_ lattice were most clearly captured at the highest scan rate of 21 Hz among these images. The periodic pattern associated with the atomic lattice was very difficult to identify if the scan rate dropped below 12 Hz, even though the typical scan rate used for AFM imaging was around 1–3 Hz. Thermal drift is a common problem for AFM systems operating under non-vacuum environments. As a result, two consecutively scanned images generally represent slightly different locations on the sample surface [79]. Typical drift velocities between the tip and the sample range from 0.25 to 8.3 Ås^−1^ [80,81,82]. An increased scan rate will result in a decreased image acquisition time, thus reducing the overall image distortion due to slow thermal drift. We illustrated the distortion caused by a constant tip drift velocity in Figure 3d. Our measured LFM images in Figure 3a–c demonstrated unambiguously that the higher the scan rate, the smaller the distortion.

##### Interplay between the Scan Size and the Scan Rate

**Figure 4:**
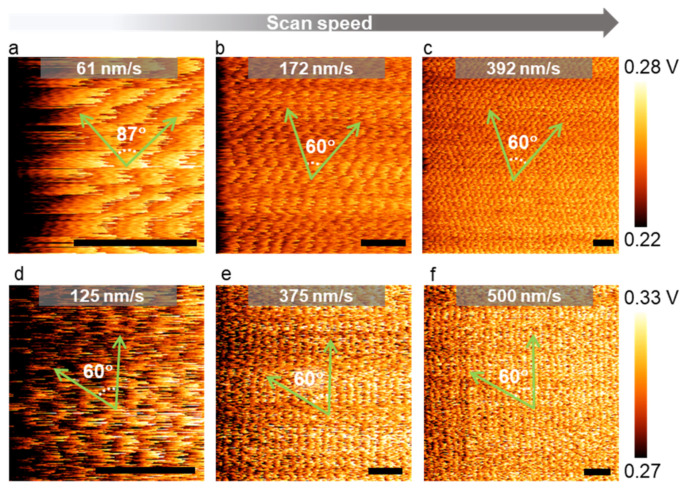
The effect of the scan speed on LFM lattice images (color range adjusted). (**a**–**c**) LFM raw data of MoSe_2_ at various scan speeds (calibrated scale bar: 1 nm); (**d**–**f**) LFM raw data of graphene at various scan speeds (scale bar: 1 nm). All green arrows are aligned along the zigzag direction.

So far, we have investigated the effects of the scan size and the scan rate separately. However, these two scan parameters are related to one another as scan speed = 2 × scan size × scan rate, when the tip moves in a raster fashion during imaging. Thus, it is possible to produce more accurate lattice images by LFM, as long as the scan speed is set high enough to compensate for the drift effect. LFM lattice images obtained from the graphene and monolayer MoS_2_ in Figure 4 clearly revealed this empirical rule. For an image containing 2*^N^* × 2*^N^* pixels, if one roundtrip line scan (trace-retrace) along the fast-scan direction (usually referred to as the *x*-axis) requires a certain amount of time ∆*t*, it takes a duration of 2*^N^* × ∆*t* for the tip to cover the equal distance in the slow-scan direction (the *y*-axis). Therefore, it is important to extract crystallographic information such as lattice constants or crystal orientation from the fast-scan direction which involves negligible distortion. The hexagonal lattice structure in Figure 4a appeared to be compressed. We attributed this kind of distortion to the difference in the actual traveling distance of the tip on the sample surface in the slow-scan direction, which depended on the relative tip−sample drift velocity along the slow-scan direction. This type of artificial image distortion can be easily confused with strain-induced lattice distortion [83,84]. One can only distinguish them by performing a careful comparison measurement on a well-defined reference sample using the same scan parameters. In addition, we noted that the sharpness of atomic images was nearly independent of the scan speed (observed in the range of 60–400 nms^−1^). The magnitude of friction is known to change with scan speed [85]. However, the sharpness of atomic images appeared to be strongly related to the saw-tooth amplitude (jump height) only (Figure S3).

##### Gain Dependence

**Figure 5:**
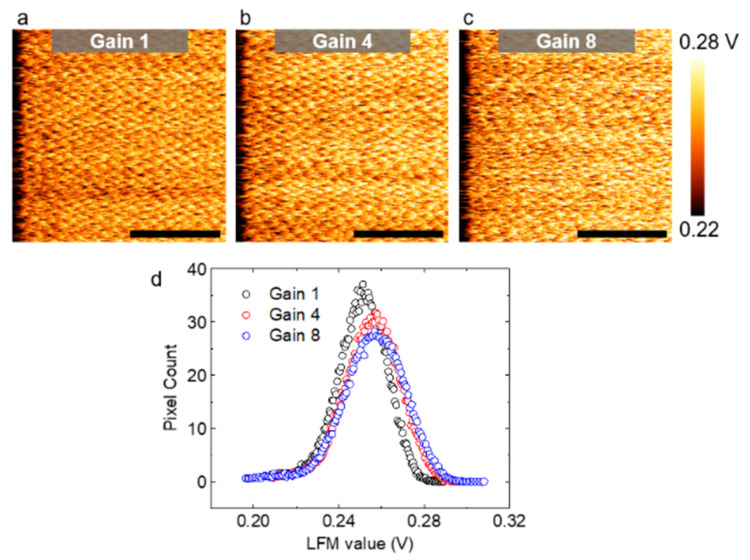
The effect of the gain on atomic-level LFM images (color range adjusted). (**a**–**c**) LFM images of multilayer graphene acquired at various gain values and at a scan rate of 19 Hz. The lattice structure became more difficult to identify, as the gain value increased. All scale bars are calibrated and represent 2 nm; (**d**) number of pixels by the LFM value for different gains. The vertical axis indicates how many pixels in an image belong to a certain LFM value. As the gain increased, the LFM data values were more spread out from the mean.

Next, we investigated the effect of feedback gain using the example of LFM images taken from multilayer graphene at 19 Hz. For a proper comparison between images, we equally set the maximum/minimum limits of each color bar. Counterintuitively, the image sharpness tended to decrease with the increasing gain (Figure 5a–c). Each pixel in Figure 5a–c represents the strength of the LFM signal. A high LFM reading in volts indicated a high friction. The number of pixels against the LFM value is plotted in Figure 5d. It can be seen in Figure 5d that the LFM data tended to be more broadly distributed as the gain increased from 1 to 4 to 8, indicating that the atomic image became dull as the gain increased. The observed gain dependence can be explained by the unavoidable mechanical crosstalk between the vertical and lateral deflections of the cantilever according to previous studies [86,87,88]. This finding is consistent with the result of a recent study in which the boundary of graphene and Si were imaged as a function of the gain with LFM. In their case, a large contrast was expected in the LFM images due to the considerable difference in the friction properties between graphene and Si (about an order of magnitude difference when measured by a Si AFM tip) [44,89]. Nevertheless, the contrast became progressively undetectable, as the gain increased [86]. In other words, if the gain was increased excessively, the friction force could not be correctly measured. Consequently, LFM images did not reveal the atomic lattice. 

##### Load Dependence

**Figure 6:**
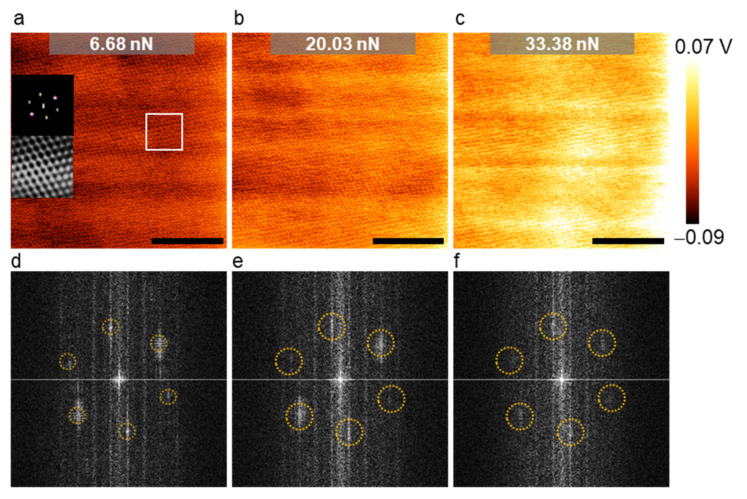
The effect of the load (i.e., setpoint) on LFM lattice images. (**a**–**c**) LFM raw data of MoS_2_ at various loads (calibrated scale bar: 5 nm; frequency: 17.1 Hz). The insets in (**a**) represent the filtered FFT images of the area enclosed by the white rectangle and its corresponding inverse FFT image; (**d**–**f**) corresponding unfiltered FFT images of MoS_2_. The six FFT spots associated with the hexagonal lattice structure are highlighted with dotted circles. The spots were less noticeable at a load of 33.38 nN, as seen in (**f**).

We compared LFM images of MoS_2_ taken at various setpoints to study the effect of the load (Figure 6a–c). In order to compare the images directly, we set the range of color bars to be the same. As expected, the friction force increased with the load; the higher the load, the brighter the image (Figure 6a–c). In addition, we found that the periodicity associated with the hexagonal atomic lattice tended to become less and less pronounced with the increasing load (Figure 6a–c). We can easily confirm from the insets in Figure 6a that the LFM image obtained at a relatively low load of 6.68 nN well represented the hexagonal lattice. We also presented the raw FFT images corresponding to the three images (Figure 6d–f). Six FFT spots representing the hexagonal lattice were visible in the raw FFT image acquired at 6.68 nN (Figure 6d). In contrast, in the FFT image obtained at 30.38 nN, only four FFT spots were visible, indicating that the lattice was deformed locally (Figure 6f). This change may be due to the increased shear force the tip exerted on the MoS_2_ monolayer during scanning as the load increased. Therefore, we recommended using a moderate load (<10 nN) to avoid this type of distortion in the atomic lattice imaging of 2D materials. However, the corrugation of the potential energy surface reduce with the load; thus, the stick−slip contrast can disappear at very low loads (in the adhesive regime) [90]. In this regard, we recommended using a positive load for image clarity, and the minimum load we used in our experiments was 2.9 nN.

##### Effect of the Substrate Roughness

**Figure 7:**
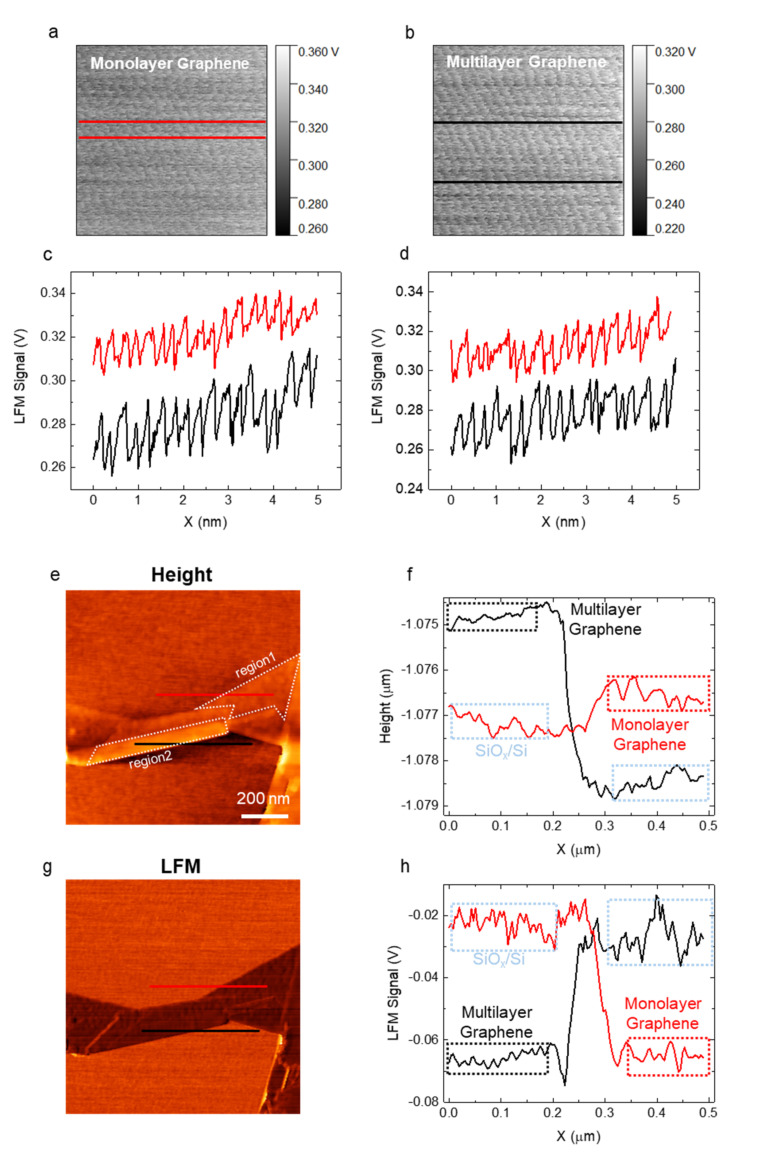
Demonstration of substrate roughness effect. (**a**) High-resolution LFM raw (unflatten and unfiltered; color range adjusted) image of monolayer graphene acquired at a scan size of 50 Å, a scan rate of 22.12 Hz, and a load of 3.4 nN; (**b**) high-resolution LFM raw (unflatten and unfiltered) image of multilayer graphene acquired at a scan size of 50 Å, a scan rate of 22.45 Hz, and a load of 2.9 nN. (**a**,**b**) were obtained using the same atomic force microscopy (AFM) probe; (**c**,**d**) comparison of cross-sectional profiles along the red (monolayer) and black (multilayer) lines in (**a**,**b**); (**e**) height image of graphene; (**f**) comparison of cross-sectional profiles along the red and black lines in (**e**); (**g**) LFM image of graphene; (**h**) comparison of cross-sectional profiles along the red and black lines in (**g**).

Lastly, we investigated the influence of the substrate roughness. We acquired dozens of images from monolayer graphene and multilayer graphene on the same Si substrate (roughness: 0.3–0.4 nm) using a single AFM tip with various combinations of scan parameters following the protocol. A representative atomic lattice image of monolayer graphene is presented in Figure 7a, and that of multilayer graphene is presented in Figure 7b. For a direct comparison, we set the range of color bars to be the same. We analyzed the images obtained by optimizing the parameters and found that, in general, the sharpness of the atomic lattice images of monolayer graphene tended to be lower than that of multilayer graphene (Figure 7a–d, including dozens of other images). The tendency may be due to the roughness of the substrate. Figure 7a,b were obtained from region1 (monolayer) and region2 (multilayer), respectively (Figure 7e). The analyses of the roughness of Si substrates, monolayer graphene, and multilayer graphene through height and LFM friction images and cross-sectional profiles showed that the roughness of the substrate directly affected the friction of monolayer graphene (Figure 7e–h). The rougher the surface, the stronger the feedback-driven tip-to-sample distance adjustment, suppressing the stick-slip phenomenon [85].

#### 3.1.2. Protocol Established Taking into Account Main Parameters

We summarized the protocol established through a series of experiments presented above in Figure S4. First, it is necessary to select an appropriate scan size, such that an image contained the desired number of lattice points. Generally, we recommend using scan sizes less than 100 Å if the lattice constant is as small as graphene. On the other hand, for TMDs, high-quality atomic lattice images can be obtained even at a scan size of 300 Å. Next, one should increase the scan rate as aforementioned until the angle between two nearest zigzag (or armchair) crystallographic axes in the image does not change with scan rate. Under common laboratory conditions, atomic lattices can be satisfactorily visualized in air by selecting a scan rate between 15 and 35 Hz, a scan size between 20 and 300 Å, a load less than 1 nN, and a gain value less than or equal to 1. The optimal scan rate varies depending on a variety of factors, including the scan size and the thermal drift velocity. When the environmental temperature fluctuations are large, we recommend using a larger scan size (>60 Å) and scan rate (>25 Hz) within the range mentioned above.

### 3.2. Application of the Protocol

#### 3.2.1. Proofs of Protocol Using Various Commercial AFMs

**Figure 8:**
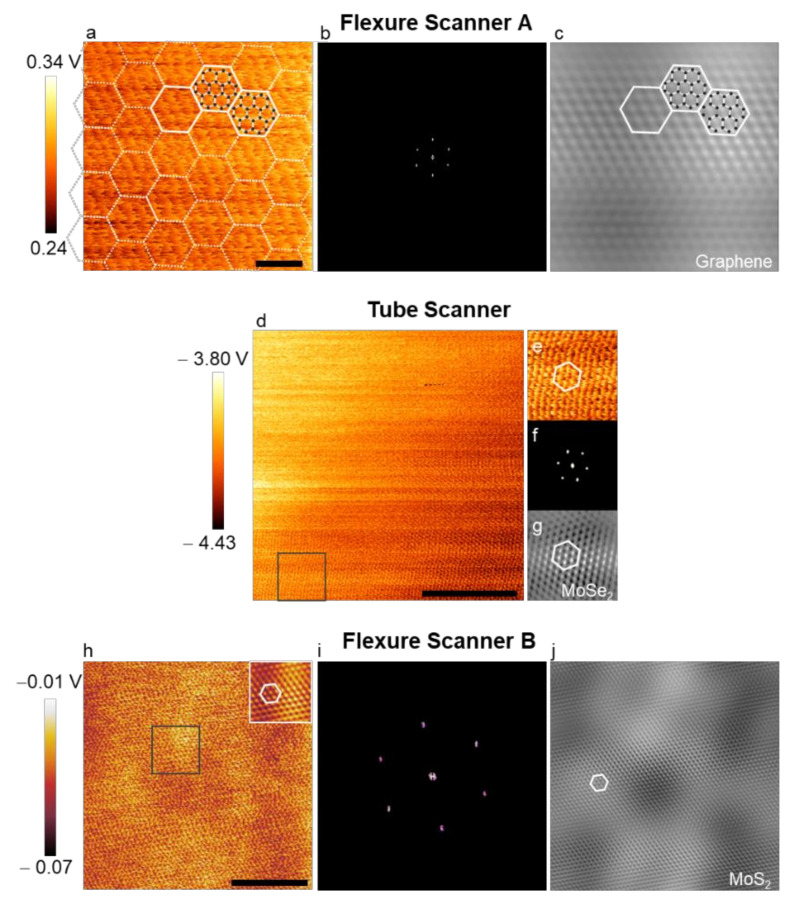
Demonstration of LFM lattice images obtained by applying our protocol to several commercial AFMs. (**a**) LFM raw (unflatten and unfiltered; color range adjusted) image of graphene acquired at a scan size of 50 Å and a scan rate of 22.45 Hz using an XE-100 AFM system. The scale bar corresponds to 1 nm; (**b**) filtered FFT image of the raw data shown in (**a**); (**c**) resulting inverse FFT image. The black dots in (**a**) and (**c**) represent carbon atoms arranged around hollow sites; (**d**) LFM raw image of MoSe_2_ collected at a scan size of 300 Å and a scan rate of 12.90 Hz using a CP-II AFM system. The scale bar corresponds to 10 nm; (**e**) the enlarged view of the squared area in (**d**); (**f**) filtered FFT image of (**e**); (**g**) the resulting inverse FFT image; (**h**–**j**) LFM image of MoS_2_ obtained at a nominal scan size of 250 Å and a scan rate of 17.0 Hz using an NX10 AFM system, the filtered FFT, and the resulting inverse FFT. The inset in (**h**) is an inverse FFT image of the squared area. The scale bar in (**h**) corresponds to 10 nm. All white hexagons are for visual guidance.

To demonstrate this protocol, we collected several atomic lattice images from graphene, MoSe_2_, and MoS_2_ using different commercial AFMs (Figure 8). The LFM raw image of graphene shown in Figure 8a was taken with a scan rate of 22.45 Hz, a scan size of 50 Å, and a 0.037 gain using an XE-100 AFM system. The filtered FFT image, including high-intensity spots arranged in a hexagonal shape (Figure 8b) and the filtered real-space image (Figure 8c), confirmed that the presented protocol can be used to visualize the graphene lattice accurately. More images obtained from MoSe_2_ and MoS_2_ monolayers and their FFT images are reported in Figure 8d–j. The LFM raw image of MoSe_2_ was taken at 12.9 Hz with a scan size of 300 Å and a gain of 1 (Figure 8d), while that of MoS_2_ was collected at 17.0 Hz with a scan size of 250 Å and a gain of 0.51 (Figure 8h) using two other AFMs. We successfully obtained well-defined local atomic lattice images under atmospheric conditions using these parameters following our protocol. In addition, their filtered FFT and inverse FFT images all showed the expected MoSe_2_ (Figure 8f,g) and MoS_2_ lattice (Figure 8i,j)-related images.

#### 3.2.2. Key Example: The Atomic Structure of Torn and Self-Folded Edges of Graphene

**Figure 9:**
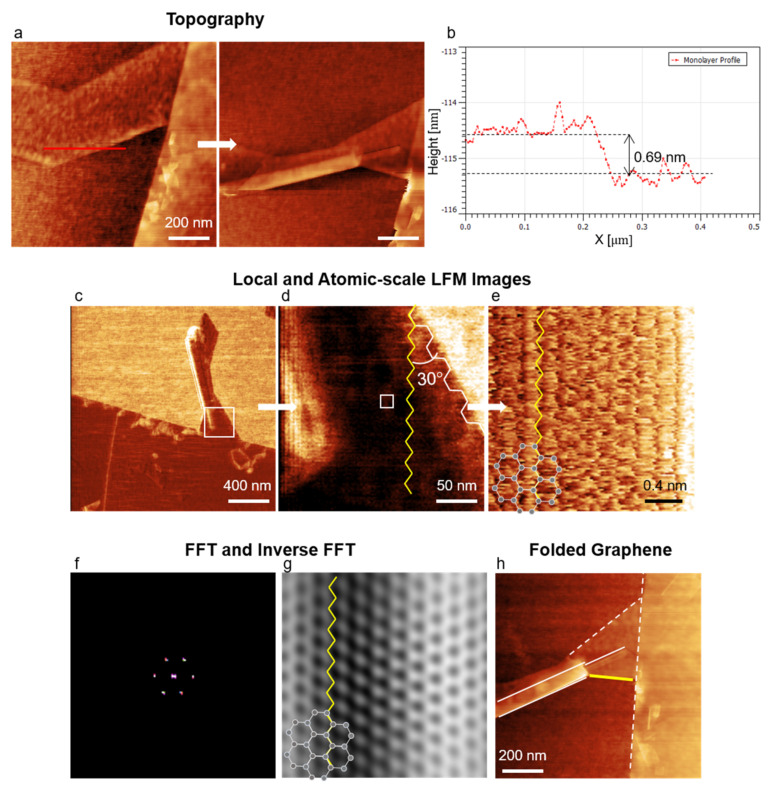
The edges of self-folded graphene nanostructure. (**a**) Topographies of graphene before and after the AFM-induced self-folding; (**b**) cross-sectional profile along the red line in (**a**); (**c**) LFM image of the folded graphene; (**d**) LFM image taken from the squared area in (**c**); (**e**) atomic-scale image collected from the white square in (**d**); (**f**) filtered FFT image of (**e**); (**g**) inverse FFT image of (**e**); (**h**) topography of the folded graphene. The white-solid (yellow-solid) line corresponds to the edge folded along the armchair (zigzag) direction. The white-dotted line is the edge torn along the armchair direction during mechanical ex-foliation. The white hexagons in (**e**,**g**) are for visual guidance.

We applied our protocol to another case. One of the most compelling applications of graphene-based materials is to enable control of their physical properties through edge engineering [91]. Thus, a nondestructive method of identifying edge structures of 2D materials in graphene-based devices is highly desirable. TEM and STM are not suitable for this task since the sample preparation process for TEM is destructive, and STM often requires the transfer of 2D materials onto a conductive substrate. We addressed this challenge by applying our protocol. We prepared monolayer and multilayer graphene using the exfoliation method [92] and applied a normal force of 13 nN onto a graphene monolayer repeatedly with an AFM tip to induce self-folding [31] several times (Figure 9a,b). We collected LFM images using our protocol to investigate the crystallographic orientation of self-folded and torn edges of the folded graphene nanostructure (Figure 9c–g).

We found that self-folding and ripping occurred primarily in the armchair direction. Out of four self-folding events, three occurred along the armchair direction, and one along the zigzag direction (solid lines; Figure 9h). Additionally, among 16 torn edges, nine were armchair edges, four were zigzag edges, and three were chiral edges (Figure S5). Our findings are consistent with the results of previous experiments [93,94]. According to several molecular dynamics simulations, the shear modulus in the armchair direction is generally smaller than that in the zigzag direction [95,96,97]. The armchair graphene shows a slightly lower strength than the zigzag graphene [96]. This difference in mechanical properties explains our experimental observations. While the experiments reported here focused on determining the crystallographic orientation of monolayers, AFM can also be used to image superlattices formed in a vdW heterostructure (e.g., moiré crystals formed by graphene/hBN) [32,48].

## 4. Conclusions

In conclusion, we have developed a protocol to obtain high-quality atomic lattice images of 2D materials using an AFM operated under the ambient environment. The challenges caused by inevitable thermal drift can be mitigated to a large extent by properly selecting several parameters, including the scan size, scan rate, and gain as well as by disenabling the XY feedback loop. The optimal values for these scan parameters varied with the drift velocity specific to a laboratory. In addition, we found that the load and the substrate roughness influenced the accuracy and the sharpness of the atomic lattice image, respectively, and we estimated the contact area and the stiffness between the AFM tip and the Si substrate using the Hertz model so that others can adapt the proposed protocol readily. Using this protocol, we have demonstrated that the atomic structure of self-folded and torn edges of graphene can be determined. Accurate atomic-scale images can be used to identify local strains and twist angle variations and guide the extensive effort to improve the quality of vdW heterostructures and 2D materials such as graphene, hBN, TMDs, MXenes, and perovskite oxides.

## Data Availability

The data presented in this study are available on request from the corresponding author.

## Supplementary Materials

The following supporting information can be downloaded at https://www.mdpi.com/article/10.3390/nano12091542/s1, Figure S1: Tip characterization; Figure S2: Before and after color range adjustment; Figure S3: Effect of the scanning speed on the image sharpness; Figure S4: AFM protocol for accurate atomic lattice imaging; Figure S5: Graphene torn-edge analysis; Table S1: Atomic lattice images of laminates obtained with the contact-mode AFM in air. References [12,28,29,30,32,33,34,35,36,37,39,40,41,43,44,45,46,47,48,49,50,51,67,68,69,98,99] are cited in the supplementary materials.
