# Supplementary material for: Accurate Atomic-Scale Imaging of Two-Dimensional Lattices Using Atomic Force Microscopy in Ambient Conditions
Source: Nanomaterials (Basel). 2022 May 2;12(9):1542. doi: 10.3390/nano12091542 (PMC9104726; doi:10.3390/nano12091542)
Supplement: Supplementary file 1 [file nanomaterials-12-01542-s001.zip › nanomaterials-1700678-supplementary.pdf]

nanomaterials

MDPI

Supplementary Materials

Accurate Atomic-Scale Imaging of Two-Dimensional Lattices Using Atomic Force Microscopy in Ambient Conditions

Sunghyun Kim [1,†], Donghyeon Moon [2,†], Bo Ram Jeon [1], Jegyeong Yeon [1], Xiaoqin Li [3,4] and Suenne Kim [2,*]

[1] Department of Applied Physics, Hanyang University, Ansan 15588, Korea; shdak6990@gmail.com (S.K.); brhu1018@gmail.com (B.R.J.); jaekyeong.yeon@gmail.com (J.Y.)
[2] Department of Photonics and Nanoelectronics, Hanyang University, Ansan 15588, Korea; dhm227@hanyang.ac.kr
[3] Department of Physics and Center for Complex Quantum Systems, The University of Texas at Austin, Austin, TX 78712, USA; elaineli@physics.utexas.edu
[4] Texas Materials Institute, The University of Texas at Austin, Austin, TX 78712, USA
* Correspondence: skim446@hanyang.ac.kr; Tel.: +82-31-400-5472
† These authors contributed equally to this work.

Table S1. Atomic lattice images of laminates obtained with the contact-mode AFM in air.

	Group	Lattice	Post-processing	Notes	Reference
Special AFMs or Tips	1	Graphite, MoS_2	N	UHV chamber N_2 environment	[1]
	2	Graphite	N	W tip, Homebuilt AFM	[2]
	3	Graphite		CNT tip	[3]
	4	Graphite		W tip, Homebuilt STM/AFM	[4,5]
	5	TaS_2	N	Super-sharp tip	[6]
	6	MoS_2	N	Symmetric design (Avoid drift, Cypher AFM)	[7]
	7	Graphene, hBN		Symmetric design (Avoid drift, Cypher AFM)	[8]
	8	MoS_2, WS_2		Symmetric design (Avoid drift, Cypher AFM)	[9]
	9	Graphene,	N	Symmetric design (Avoid drift, Cypher AFM)	[10]
	10	Mica (> 0.5 nm)	Y	Small scanner	[11]
	11	Graphite, Mica, MoS_2	N	Sample cleaving critical	[12]
	12	Graphite	N	Sample cleaving critical	[13]
	13	$NbSe_2$	N	Sample cleaving critical	[14]
	14	Graphene, Multilayer G	N		[15]
		Graphene	Y		[16]
	15	Graphene	N		[17]
			Y		[18]
	16	Graphene			[19]
	17	Graphene, hBN	Y	Small scanner	[20]

Excluding atomic images obtained via electrical signals using AFM techniques that require conductive substrate (ex. Piezoresponse Force Microscopy). Excluding exemplary images advertised by commercial AFM companies. For a group that has published multiple papers, only the representative papers are referenced.

Atomic-lattice images of 2D materials acquired using the contact-mode AFM in air have been provided by only a few groups worldwide (Table S1). Most atomic images taken in the ambient environment have been obtained by AFM experts using special homebuilt STM-AFM, functionalized tips, carbon nanotube tips, or symmetrically-designed Cypher AFM (exceptionally low noise floor < 15 pm [21]); but their uptake is limited. No systematic investigations or careful protocol have been provided so far.

Miscellaneous

Tip Characterization

Interestingly, we found that sharp tips are not critical for obtaining atomic lattice images during imaging following the proposed protocol. Atomic lattice images were acquired repeatedly at low loads (< 10 nN) even after the tip was pressed to ~30 nN to obtain Figure 6c. Although the sharpness of the tip is considered important [22-24], it is reported that regular periodicity of the sample can be obtained even with a tip with multiple contacts[6].

The shape of the NSC36C AFM tip used for imaging dozens of times is shown in Figure S1. We use the NT-MDT TGT1 silicon calibration grating consisting of an array of silicon spikes with heights of 0.3 μm or more and tip curvature radii smaller than 10 nm. A contact mode height image is presented in Figure S1a. The cross-sectional profile along the line shows an apex diameter of ~92.2±5 nm (Figures S1b and S1c). Taking the convolution effect into account, by subtracting the nominal diameter of the spike (~20 nm), we find that this tip has an apparent diameter of ~72.2±5 nm.

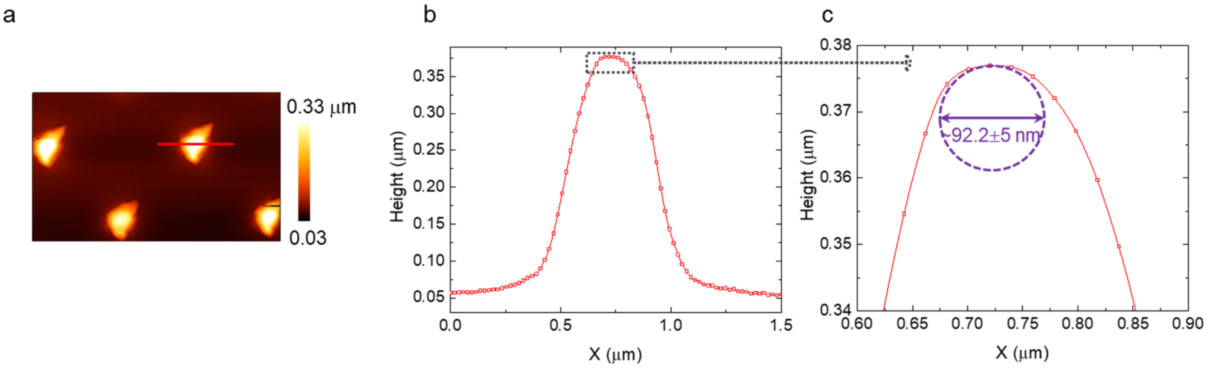

Figure S1. Tip characterization. (**a**) Contact mode height image of TGT01 grating; (**b**) Cross-sectional profile of a calibration Si spike obtained using an AFM tip; (**c**) Magnified cross-sectional profile.

Determination of the Contact Stiffness

The contact stiffness between a medium sharp Si tip (1.43 Nm^{-1}) and the reference Si substrate at the load of 10.3 nN is deduced in dry atmosphere (~23±2 RH%) from the Hertz model[25,26]. The tip-sample system can be modeled as a sphere pressing a flat surface. In this case, the contact stiffness ($k_{contact}$) is directly proportional to the contact radius a.

$$k_{\mathrm{contact}} = 8G^{*}a \quad (1)$$

where $G^{*} = \left[(2-\nu_{\mathrm{tip}})G_{\mathrm{tip}}{}^{-1} + (2-\nu_{\mathrm{sample}})G_{\mathrm{sample}}{}^{-1}\right]$, $a = [3RL(4E^{*})^{-1}]^{1/3}$, $G = E[2(1+\nu)]^{-1}$, $E^{*} = \left[(1-\nu_{\mathrm{tip}}{}^{2})E_{\mathrm{tip}}{}^{-1} + (1-\nu_{\mathrm{sample}}{}^{2})E_{\mathrm{sample}}{}^{-1}\right]$; G_{tip} and G_{sample}: the shear moduli ($E_{\mathrm{tip}} = E_{\mathrm{sample}} = 130$ GPa, Si (100)[27]); ν_{tip} and ν_{sample}: the Poisson's ratios ($\nu_{\mathrm{tip}} = \nu_{\mathrm{sample}} = 0.28$); R: the radius of curvature (72.2 nm); L: the load (10.3 nN).

Using the values above, we estimate the contact radius, *a,* to be 2.04 nm and $k_{contact}$ to be 240.9 N/m.

Color Range Adjustment

After adjusting the color range of the image, the atomic lattice structure appears sharper.

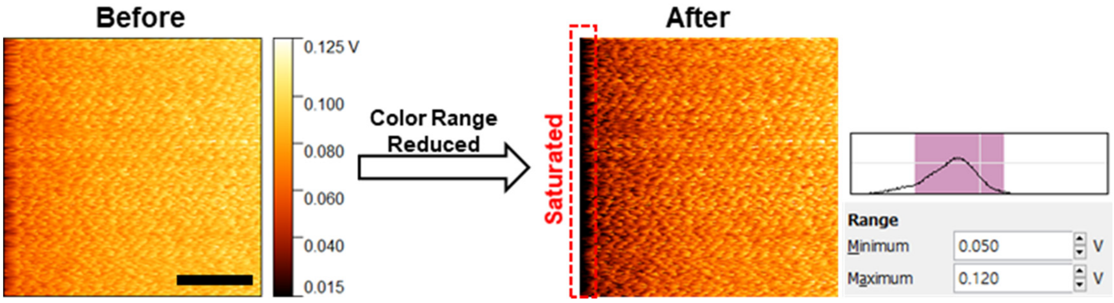

Figure S2. Before and after color range adjustment. LFM trace images of a MoS_2 monolayer before and after color range adjustment (calibrated scale bar: 2 nm, scan rate: 21 Hz).

Effect of speed on image sharpness

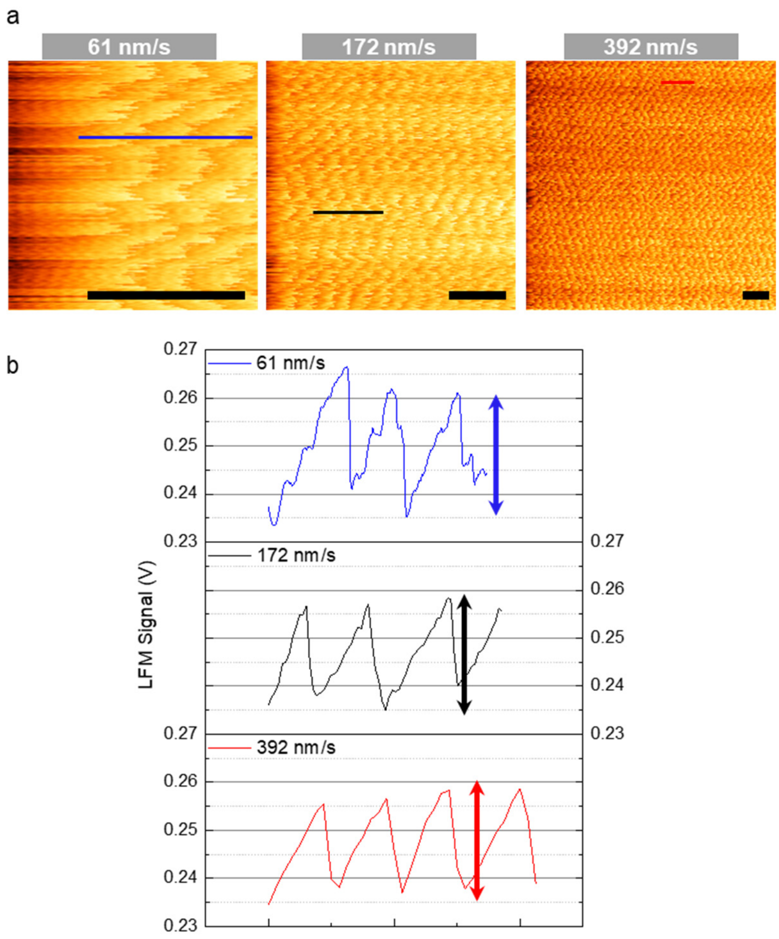

Figure S3. Effect of scanning speed on image sharpness. (**a**) LFM raw images of MoSe2 consecutively obtained in air at 61, 172, and 392 nms-1 (calibrated scale bar: 1 nm); (**b**) Cross-sectional profiles along the blue, black, and red lines. The saw-tooth amplitude (jump height) is similar at 20 mV in all three cases.

Protocol

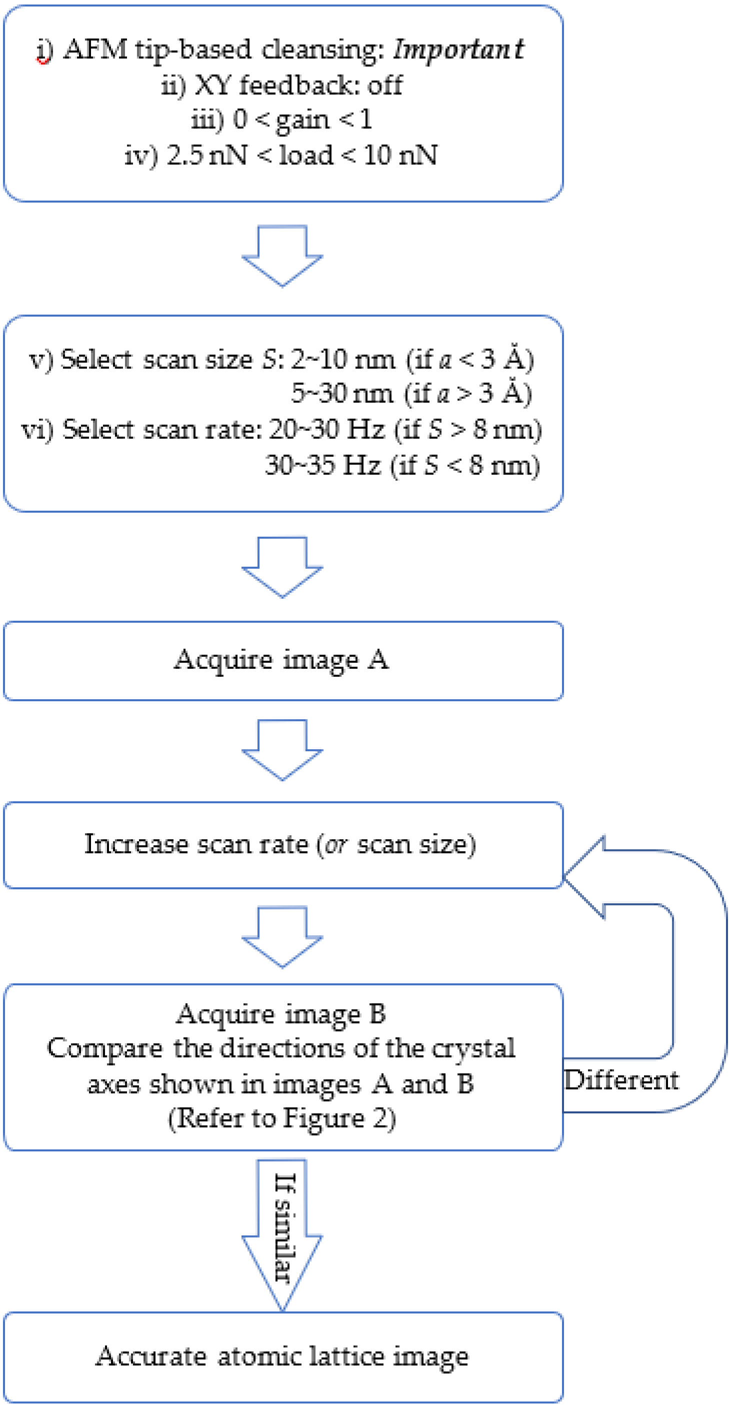

Figure S4. AFM protocol for accurate atomic lattice imaging. The protocol allows one to obtain accurate atomic lattice images in air using any commercial AFM.

Additional Application-Atomic Structures of Torn Edges

The torn edges resulting from mechanical exfoliation of highly ordered pyrolytic graphite (HOPG) were investigated. Most of the straight edges were armchair or zigzag edges (Figure S5). The consistency between our and previous studies supports the reliability of the proposed protocol.

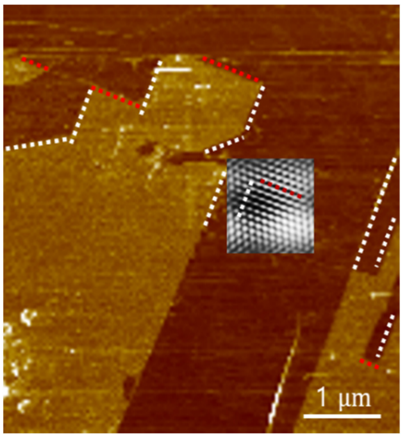

Figure S5. Graphene torn-edge analysis. An LFM image of mechanically-exfoliated graphene. The inset shows an inverse FFT image obtained from a nearby region. The white-dotted (red-dotted) lines correspond to the edges ripped along the armchair (zigzag) direction.

References

1. Lee, C.; Li, Q.; Kalb, W.; Liu, X.-Z.; Berger, H.; Carpick, R.W.; Hone, J. Frictional Characteristics of Atomically Thin Sheets. *Science* **2010**, *328*, 76–80. https://doi.org/10.1126/science.1184167.
2. Mate, C.M.; McClelland, G.M.; Erlandsson, R.; Chiang, S. Atomic-scale friction of a tungsten tip on a graphite surface. *Phys. Rev. Lett.* **1987**, *59*, 1942–1945. https://doi.org/10.1103/PhysRevLett.59.1942.
3. Lai, W.-C.; Chin, S.-C.; Chang, Y.-C.; Chen, L.-Y.; Chang, C.-S. Lattice-resolved frictional pattern probed by tailored carbon nanotubes. *Nanotechnology* **2009**, *21*, 055702. https://doi.org/10.1088/0957-4484/21/5/055702.
4. Sugawara, Y.; Ishizaka, T.; Morita, S. Scanning force/tunneling microscopy of a graphite surface in air. *Journal of Vacuum Science & Technology B: Microelectronics and Nanometer Structures Processing, Measurement, and Phenomena* **1991**, *9*, 1092–1095. https://doi.org/10.1116/1.585267.
5. Sugawara, Y.; Ishizaka, T.; Morita, S.; Imai, S.; Mikoshiba, N. Simultaneous Observation of Atomically Resolved AFM/STM Images of a Graphite Surface. *Jpn. J. Appl. Phys.* **1990**, *29*, L157-L159. https://doi.org/10.1143/jjap.29.l157.
6. Sheiko, S.S.; Möller, M.; Reuvekamp, E.M.C.M.; Zandbergen, H.W. Calibration and evaluation of scanning-force-microscopy probes. *Phys. Rev. B* **1993**, *48*, 5675–5678. https://doi.org/10.1103/PhysRevB.48.5675.
7. Acikgoz, O.; Baykara, M.Z. Speed dependence of friction on single-layer and bulk MoS2 measured by atomic force microscopy. *Appl. Phys. Lett.* **2020**, *116*, 071603. https://doi.org/10.1063/1.5142712.
8. Summerfield, A.; Davies, A.; Cheng, T.S.; Korolkov, V.V.; Cho, Y.; Mellor, C.J.; Foxon, C.T.; Khlobystov, A.N.; Watanabe, K.; Taniguchi, T.; et al. Strain-Engineered Graphene Grown on Hexagonal Boron Nitride by Molecular Beam Epitaxy. *Sci. Rep.* **2016**, *6*, 22440. https://doi.org/10.1038/srep22440.
9. Zhou, X.; Liu, Y.; Hu, X.; Fang, L.; Song, Y.; Liu, D.; Luo, J. Influence of elastic property on the friction between atomic force microscope tips and 2D materials. *Nanotechnology* **2020**, *31*, 285710. https://doi.org/10.1088/1361-6528/ab7f7d.
10. Marsden, A.J.; Phillips, M.; Wilson, N.R. Friction force microscopy: A simple technique for identifying graphene on rough substrates and mapping the orientation of graphene grains on copper. *Nanotechnology* **2013**, *24*, 255704. https://doi.org/10.1088/0957-4484/24/25/255704.
11. Gupta, V.; Hampton, M.A.; Nguyen, A.V.; Miller, J.D. Crystal lattice imaging of the silica and alumina faces of kaolinite using atomic force microscopy. *J. Colloid Interface Sci.* **2010**, *352*, 75–80. https://doi.org/10.1016/j.jcis.2010.08.002.
12. Sumomogi, T.; Hieda, K.; Endo, T.; Kuwahara, K. Influence of atmosphere humidity on tribological properties in scanning probe microscope observation. *Appl. Phys. A* **1998**, *66*, S299-S303. https://doi.org/10.1007/s003390051150.
13. Ruan, J.A.; Bhushan, B. Atomic-scale and microscale friction studies of graphite and diamond using friction force microscopy. *J. Appl. Phys.* **1994**, *76*, 5022–5035. https://doi.org/10.1063/1.357214.
14. Schimmel, T.; Koch, T.; Küppers, J.; Lux-Steiner, M. True atomic resolution under ambient conditions obtained by atomic force microscopy in the contact mode. *Appl. Phys. A* **1999**, *68*, 399–402. https://doi.org/10.1007/s003390050912.
15. Liu, Z.; Ma, T.; Liu, L. Optical-assistant characterization of friction anisotropy properties of single-crystal graphene domains. *Tribol. Int.* **2017**, *110*, 131–139. https://doi.org/10.1016/j.triboint.2017.02.027.
16. Zhang, Y.; Yu, F.; Li, G.; Liu, L.; Liu, G.; Zhang, Z.; Wang, Y.; Wejinya, U.C.; Xi, N. Online Determination of Graphene Lattice Orientation Through Lateral Forces. *Nanoscale Res. Lett.* **2016**, *11*, 353. https://doi.org/10.1186/s11671-016-1553-z.
17. Almeida, C.M.; Prioli, R.; Fragneaud, B.; Cançado, L.G.; Paupitz, R.; Galvão, D.S.; De Cicco, M.; Menezes, M.G.; Achete, C.A.; Capaz, R.B. Giant and Tunable Anisotropy of Nanoscale Friction in Graphene. *Sci. Rep.* **2016**, *6*, 31569. https://doi.org/10.1038/srep31569.
18. Almeida, C.M.; Carozo, V.; Prioli, R.; Achete, C.A. Identification of graphene crystallographic orientation by atomic force microscopy. *Journal of Applied Physics* **2011**, *110*, 086101. https://doi.org/10.1063/1.3642991.

19. Zambudio, A.; Gnecco, E.; Colchero, J.; Pérez, R.; Gómez-Herrero, J.; Gómez-Navarro, C. Fine defect engineering of graphene friction. *Carbon* **2021**, *182*, 735–741. https://doi.org/10.1016/j.carbon.2021.06.064.
20. Tang, S.; Wang, H.; Zhang, Y.; Li, A.; Xie, H.; Liu, X.; Liu, L.; Li, T.; Huang, F.; Xie, X.; et al. Precisely aligned graphene grown on hexagonal boron nitride by catalyst free chemical vapor deposition. *Sci. Rep.* **2013**, *3*, 2666. https://doi.org/10.1038/srep02666.
21. Hurley, D.; Ohler, B. White Paper: Characterizing the atomic lattices of 2D crystals with AFM. *MRS Bull.* **2017**, *42*, 89–90. https://doi.org/10.1557/mrs.2017.13.
22. Gan, Y. Atomic and subnanometer resolution in ambient conditions by atomic force microscopy. *Surf. Sci. Rep.* **2009**, *64*, 99–121. https://doi.org/10.1016/j.surfrep.2008.12.001.
23. Giessibl, F.J. AFM's path to atomic resolution. *Mater. Today* **2005**, *8*, 32–41. https://doi.org/10.1016/S1369-7021(05)00844-8.
24. Gupta, V. Crystal Lattice Imaging Using Atomic Force Microscopy. In *Atomic Force Microscopy—Imaging, Measuring and Manipulating Surfaces at the Atomic Scale*; Bellitto, V., Ed.; IntechOpen: London, UK, 2012.
25. Carpick, R.W.; Ogletree, D.F.; Salmeron, M. Lateral stiffness: A new nanomechanical measurement for the determination of shear strengths with friction force microscopy. **1997**, *70*, 1548–1550. https://doi.org/10.1063/1.118639.
26. Johnson, K.L. *Contact Mechanics*; Cambridge University Press: Cambridge, MA, USA, 1985.
27. Hopcroft, M.A.; Nix, W.D.; Kenny, T.W. What is the Young's Modulus of Silicon? *J. Microelectromechanical Syst.* **2010**, *19*, 229–238. https://doi.org/10.1109/JMEMS.2009.2039697.
